# Changes in the cellular proteins of A549 infected with Hepatitis E virus by proteomics analysis

**DOI:** 10.1186/s12917-014-0188-5

**Published:** 2014-08-30

**Authors:** Quan Shen, Yingyan Pu, Xingli Fu, Ying Xie, Xiaobo Bian, Shixing Yang, Yan Yang, Li Cui, Xiaochun Wang, Hua Wang, Wen Zhang

**Affiliations:** 1 School of Medicine, Jiangsu University, 301 Xuefu Road, Zhenjiang 212013, Jiangsu, PR China; 2 Institute of Neuroscience and Key Laboratory of Molecular Neurobiology of Ministry of Education, Second Military Medical University, Shanghai 200433, PR China; 3 The Fourth Affiliated Hospital of Jiangsu University, 20 Zhengdong Road, Zhenjiang 212001, Jiangsu, PR China; 4 School of Agriculture and Biology, Shanghai JiaoTong University, 800 Dongchuan Road, Shanghai 200240, PR China

**Keywords:** Hepatitis E Virus, Cellular proteins, Proteomics

## Abstract

**Background:**

Our understanding of Hepatitis E virus (HEV) has changed enormously over the past 30 years, from a waterborne infection causing outbreaks of acute hepatitis in developing countries to an infection of global distribution causing a range of hepatic and extra-hepatic illness. However, the key proteins playing important parts in the virus infection were still unknown. Understanding the changes of cellular proteins in these cells exposed to HEV is helpful for elucidating molecular mechanisms associated with function alterations of HEV-infected susceptible cells. In the present study, a comparative gel-based proteomic analysis was employed to study the changes in cellular proteins of A549 exposed to HEV in vitro to provide novel information for understanding the functional alterations of A549 induced by HEV infection.

**Result:**

Of 2 585-3 152 protein spots visualized on each gel using silver staining, a total of 31 protein spots were found to be differentially expressed in HEV-infected A549 cells compared with mock-infected A549, including 10 significantly up-regulated protein spots and 21 significantly down-regulated protein spots.

**Conclusion:**

Our work is the first time regarding the proteomic analysis on the cellular responses to HEV infection. This work is helpful for investigating the molecular basis associated with the interaction between HEV and the host cells although more efforts should be required to discover the mechanisms.

## Background

Hepatitis E virus (HEV), the causative agent of what has been referred to as enterically transmitted non-A, non-B hepatitis or “waterborne hepatitis”, is a major cause of epidemic hepatitis [1],[2]. Hepatitis E virus infection is known to cause waterborne epidemics and sporadic infections in developing countries [3]-[7]. Recently, there have been several reports on zoonotic foodborne autochthonous infections of hepatitis E in developed countries [8]-[12]. HEV belongs to the genus Hepevirus in the Hepeviridae family. The family also includes closely related viruses that infect pigs, rabbits, rats, deer and mongoose, which belong to the same genus as the human HEV, and the more distant avian HEV [13]. HEV is a small nonenveloped virus with a size of 27-34 nm, and has a positive-sense, single-stranded RNA genome of approximately 7.2 kb, which is capped at the 5′ termini and polyadenylated at the 3′termini [14]. The HEV genome contains three open reading frames (ORFs). ORF1 encodes a protein of 1693 amino acids containing functional motifs and domains present in the non-structural proteins of other positive-stranded RNA viruses. These functional domains include methyl transferase, protease, RNA helicase, and RNA-dependent RNA polymerase, which were important for viral replication [15]. ORF2 encodes the viral capsid protein of 660 amino acids consists of three linear domains and forms homodimers that is responsible for virion assembly, interaction with target cells, and immunogenicity [16],[17]. ORF3, which overlaps ORF2 partially, encodes a small protein of 114 amino acids involved in virion morphogenesis and viral egress from infected cells [18].Based on sequence analysis, HEV strains have been classified into four major genotypes (1-4), of which genotype 1 and 2 strains are restricted to humans, whereas genotypes 3 and 4 have a broader host range and are zoonotic [19]. Culture systems are essential for a better understanding of the biology of HEV including the interaction between virus and host cells, and potential targets for the development of antiviral drugs. Recently, an efficient culture system for HEV genotype 3 was developed for the first time with PLC/PRF/5 hepatic carcinoma cell lines and A549 lung carcinoma cell lines and faecal samples from a patient with acute hepatitis in Japan containing a very high titer of HEV RNA, though HEV has been proved to be difficult to cultivate. These cell lines also permitted to HEV genotype 4, as well as genotype 1. The establishment of cell culture system provides a convenient way to understand the unknown issues especially the mechanism of HEV replication. To date, proteomic approaches, coupling two-dimensional electrophoresis (2-DE) and mass spectrometry (MS), have been widely used to study the mechanisms of viral infection through the comparative analysis of cellular protein profiles [20],[21].

In the present study, a comparative gel-based proteomic analysis was employed to analyze the changes in cellular proteins of A549 exposed to HEV in vitro to provide novel information for understanding the functional alterations of A549 induced by HEV infection.

## Methods

### Virus

HEV positive swine fecal sample was from an experimentally infected pig with a genotype 4 HEV strain (GenBank accession no.: EF570133) with a titer of 10^3^-10^4^ pfu/mL [22]. This sample was proved to be negative for PEVs (including PTV and PEV1-10), haemagglutinating encephalomyelitis virus, Aujeszky’s disease virus, porcine circovirus type 2, porcine reproductive and respiratory syndrome virus, classical swine fever virus, Japanese encephalitis virus, porcine transmissible gastroenteritis virus, porcine epidemic diarrhoea virus, porcine rotavirus, porcine sapovirus, cytomegalovirus, porcine Torque-Teno virus and porcine parvovirus by RT-PCR or PCR methods [22]. The fecal sample was converted to 10% (w/v) suspensions in PBS (pH7.4) and clarified by centrifugation at 15, 000 g for 30 min. Supernatants were purified by passage through 0.22 μm microfilters (Millex-GV, Millipore, Japan) before viral inoculation. The HEV negative swine fecal samples were treated in the same way as control.

### Cell-culture

A549 cells were grown in Dulbecco’s modified Eagle’s medium (DMEM), supplemented with 10 %(v/v) heat-inactivated fetal calf serum (FCS, Gibico, Australia), 100 U penicillin G/ml, 100 μg streptomycin/ml and 2.5 μg amphotericin/ml, at 37°C in a humidified 5% CO_2_ atmosphere. For virus inoculation, confluent cells were trypsinized and diluted 1:4 in medium and 2.0 ml was added to six-well microplate (Corning, USA) 1 or 2 days before virus infection.

### Virus-inoculation

Monolayers of A549 cells in a six-well microplate were washed three times with PBS containing 0.2% (w/v) BSA (Sigma-Aldrich, USA) and 0.2 ml of the filtered virus stock was inoculated on the cells at room temperature. One hour after inoculation, supernatants were removed and 2 ml of maintenance medium was added. The maintenance medium consisted of 50% DMEM and 50% medium 199 (Invitrogen, USA) containing 2% (v/v) heat-inactivated FCS and 30 mM MgCl_2_ at final concentration. Culturing situation was the same as cell-culture descripted above. After inoculation, the cells were washed five times with PBS and 2 ml maintenance medium was then added. The culture medium was removed and the cells were collected after the appearance of cytopathic effect (CPE). Two-Dimensional Gel Electrophoresis (2-DE) was performed after the detection of HEV RNA and viral particles by RT-PCR, Dot-blot assay and immune electron microscopy (IEM) methods, respectively. The ultrastructal changes of infected cells were observed by transmission electron microscopy (TEM).

### Detection of HEV RNA

HEV RNA was detected by RT-PCR according to the method described previously [22]. The PCR products were analyzed in a 1.5% agarose gel, followed by staining with ethidium bromide (0.5 μg/ml) and visualization under UV light.

### Dot-Blot

For detection of HEV capsid proteins, the cells infected with HEV were lysed and 50 μg of cell lysate was loaded onto the PVDF membrane (Millipore, Bedford, MA) which was soaked in TBST buffer for 5 min in advance. The membrane was immersed in Tris-buffered saline (TBST) [10 mM Tris/HCl, pH 7.5, 0.15 M NaCl, 0.1% (v/v) Tween-20] containing 5% BSA (Amersham Biosciences, CA) and, after washing with TBST, incubated at room temperature for 1 h with 1 μg of anti-HEV ORF2 rabbit monoclonal antibody that had been raised against the recombinant HEV ORF2 protein as the primary antibody (Abcam, USA). After washing, the membrane was incubated with ECL anti-rabbit IgG, horseradish peroxidase-linked species-specific whole antibody from goat (1:2500; Amersham Biosciences) and was examined using a chemical luminescence system (ECL Western blotting detection reagents and analysis system, Bio-Rad).

### Transmission electron microscopy

After the appearance of CPE, medium was removed and the cells were collected for investigation of the ultrastructal changes by TEM. In brief, cells were collected by centrifugation and washed with 0.05 mol/L of PBS. The samples were transferred to fresh 0.5% glutaraldehyde, and kept for 30 min at 4°C, centrifuged at 12, 000 g, and fixed in 3% glutaraldehyde for 2 h. Cells were further fixed with 1% OSO_4_, dehydrated in gradually increased acetone solutions, and were embedded in epoxy resin (Epon812). Ultrathin sections were cut and stained with uranyl acetate and lead citrate. TEM were taken with a JEM–1011(Tokio, Japan) transmission electron microscope at 80 kV. The samples were viewed under TEM (Model 100 CX, JEOL, Japan) at an opening voltage of 60 kV with 40, 000 ×.

### Unlabeled immune electron microscopy

To observe the HEV particles by unlabeled immune electron microscopy, the infected cells were collected and lysed as described above. Two hundred microliter of specific of human anti-HEV ORF2 serum was added to the 500 μl of cell lysate and incubated at room temperature for 1 h followed by centrifuged at 15, 000 g for 15 min. the pellet was resolved in 20 μl of H_2_O ant then stained with 2% of phosphotungstic acid. One drop of the sample was placed onto 300-mesh grid and viewed under TEM at an opening voltage of 100 kV with 40, 000 ×.

### Sample preparation for 2-DE

The collected cells were lysed with lysis buffer (7 M urea, 2 M thiourea, 4% CHAPS) containing protease inhibitor cocktail (complete, mini, Roche, Germany). After lysing by sonication for 72 s with 38% power output, DNase I, RNase A and IPG buffer 4-7 (GE Healthcare) were supplemented into the lysis buffer. The suspensions were then incubated in the buffer for 2 h at 4°C. After removal of cellular debris by centrifugation at 12, 000 g for 30 min at 4°C, the supernatants were collected and stored at -80°C until use. The protein concentration was determined by the Plus-One 2-D Quant Kit (GE Healthcare).

### 2-DE

2-D Gel Electrophoresis was performed as previously described [20].

Briefly, approximately 100 μg of whole HeLa cell lysate was mixed with a rehydration solution containing 7 M urea, 2 M thiourea, 2% CHAPS (w/v), 1% DTT (w/v) and 0.5% IPG-buffer. The sample mixture was loaded onto pH 4-7 IPG, 17 cm strips (GE Healthcare).

IPG strips were rehydrated for 13 h at room temperature with passive rehydration.

Focusing was carried out at 20°C with the current limited to 50 μA/strip using IPGPhor II (GE Healthcare). The program was performed as follows: 100 V for 1.5 h, 250 V for 0.5 h, 500 V for 0.5 h, 1000 V for 0.5 h, 3000 V for 0.5 h, 5000 V for 0.5 h, gradient ramping to 8000 V for 2 h, then 8000 V for a total of 100000 Vh. Equilibration steps were applied immediately after focusing with equilibration buffer (6 M urea, 50 mM Tris, 2% w/v SDS, 30% v/v glycerol, and a trace of bromophenol blue, pH 8.8), containing 1% w/v DTT for 20 min, followed by incubation in the same buffer containing 2.5% w/v iodoacetamide for another 20 min. The equilibrated strip was applied directly to 12% SDS polyacrylamide gels (SDS-PAGE). The second dimension was carried out on an Ettan Dalt six (GE Healthcare) at 3 W/gel for 1 h and then at 17 W/gel for about 12 h at 16°C. The gels were stained by the modified silver staining method compatible with MS.21. Three independent cell cultures at each time point were conducted for biological replicates.

### Image acquisition and analysis

After 2D electrophoresis, the gels were scanned on a Typhoon Trio image scanner (GE Healthcare) according to the manufacturer’s protocol at a resolution of 400 dpi for each gel. After cropping with ImageQuant software (GE Healthcare), the images were subjected to automated difference-in-gel analysis and biological variation analysis (BVA) using Decyder version 6.5 software (GE Healthcare). Only the significantly differentially expressed protein spots (*P* < 0.05) with 1.5-fold different intensity or more were selected and subjected to identification by MS.

### In-Gel digestion

Protein spots of interest were manually excised from the silver-stained gels and then washed with 100 μl 50% v/v acetonile (ACN) in 25 mM ammonium bicarbonate for 1 h. After dehydration with 100% v/v ACN for 20 min, the gel pieces were thoroughly dried in a SpeedVac concentrator (Thermo Savant, U.S.A.) for 30 min. The dried gel particles were rehydrated for 45 min at 4°C with 2 μl trypsin (promega, Madison, WI) in 25 mM ammonium bicarbonate, and then incubated for 12 h at 37°C. The resulting peptides were extracted three times by 8 μl aliquots of 5% trifluoroacetic acid (TFA) in 50% ACN for 1 h at 37°C, and dried in a vacuum centrifugation.

### Mass spectrometry analysis and database search

Mass spectrometry was undertaken commercially by the Research Centre for Proteome Analysis, Shanghai Institutes for Biological Sciences, Chinese Academy of Sciences, Shanghai, China. In brief, band was subjected to tryptic digestion for 16 h followed by Matrix Assisted Laser Desorption Ionization (MALDI) mass spectrometry and was performed with a Micromass Maldi Time of Flight (MALDI-TOF) Mass Spectrometer. Spectra were acquired in the mass range 400-2000 Da. Spectra were searched against Suina Protein Data Bank in NCBI.

### Western blot analysis

Equivalent amount of proteins were separated by electrophoresis on 12% (w/v) SDS-PAGE. The fractionated proteins were then transferred electrophoretically to a PVDF membrane and blocked with TBS-T containing 5% BSA at 4°C for 2 h. The membranes were stained with rabbit anti-HSP70 polyclonal antibody (Biosynthesis, Beijing, China) at 1:1000 dilutions, rabbit anti-MAPK4 polyclonal antibody (Yopebio, Shanghai, China) at 1:1000 dilution overnight at 4°C, respectively. After washing, the membranes were immersed in HRP-conjuncted Mouse Anti-rabbit IgG antibody at 1:1 000 dilution for 2 h at 4°C. Immunoreactive protein bands were visualized with a TMB Substrate and the images were acquired.

## Results

### Detection of virus particles and antigens in infected cells

**Figure 1:**
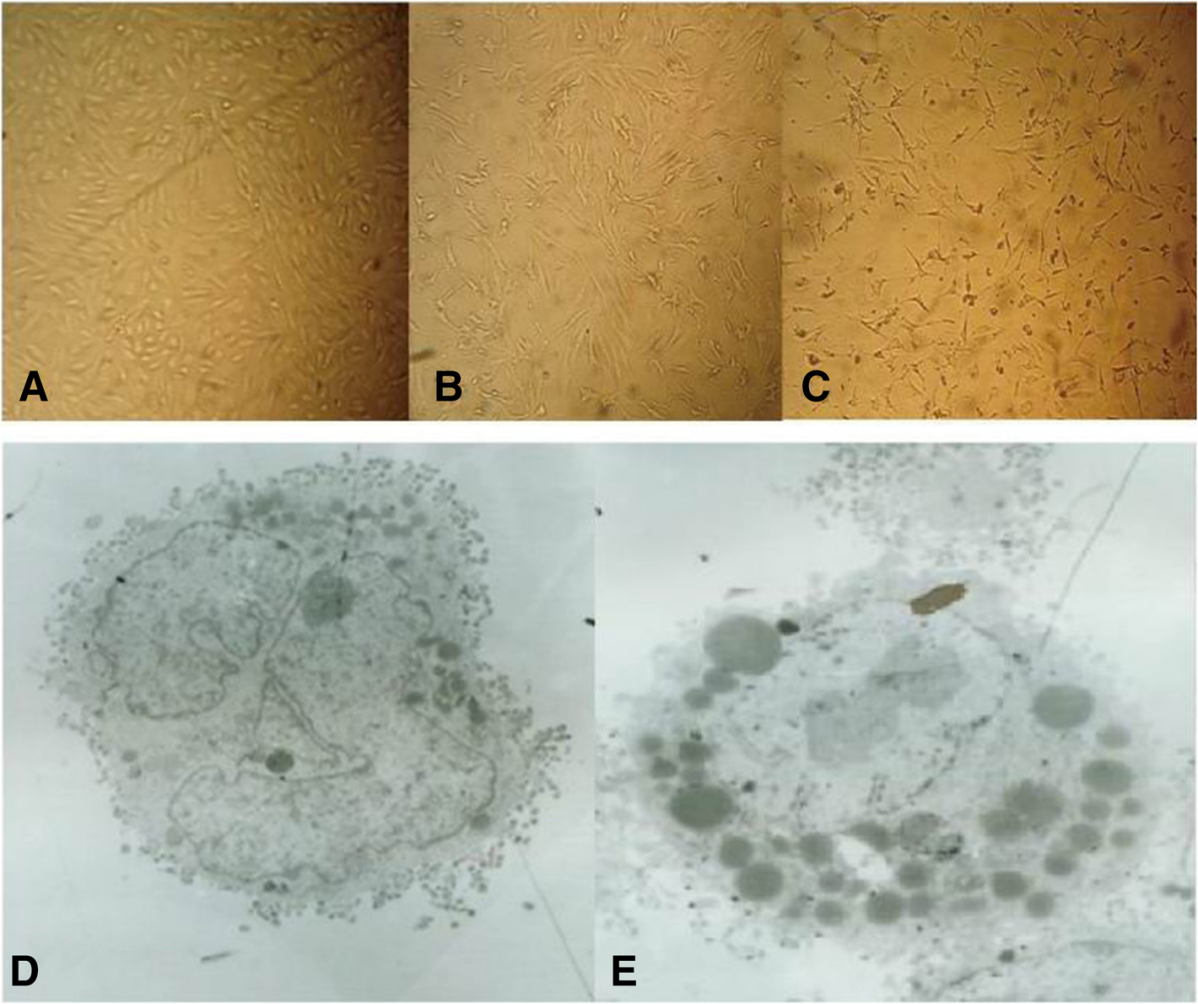
The CEP of A549 cells at 24 h (B) or on day 6 (C) post-infection, cells treated with HEV negative swine fecal sample as control (A); (blow) the structural changes of A549 cells infected with HEV were investigated by TEM (40,000 ×) (D), cells treated with HEV negative swine fecal sample as control (E).

**Figure 2:**
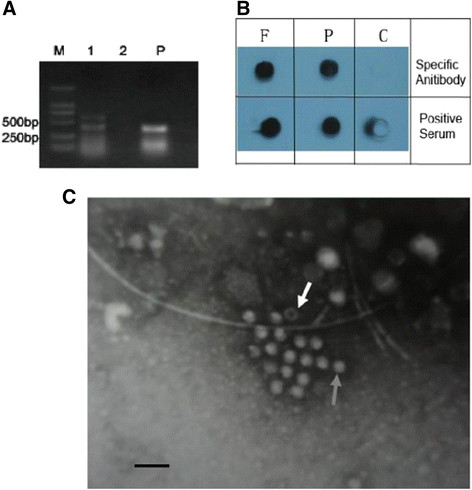
**Detection of HEV RNA (A), Virus Particles (B) and Antigens (C) in infected cells with RT-PCR, Dot-blot and unlabeled immune Electron Microscopy, respectively. A**: 1, infected A549 cells. 2, HEV negative fecal sample inoculated cells. P, HEV positive fecal sample as control; **B**: F, HEV positive fecal sample. P, HEV inoculated A549 cells. **C**, HEV negative fecal sample inoculated cells; **C,** black arrow indicated complete virus particle and white arrow indicated empty virus particle.

Visible CPE was observed at 24 h post-infection, and 90% CPE appeared on day 6 post-infection (Figure 1A-C). Structural changes of infected cells were investigated by TEM. Results showed that the number of lysosome increased, which is an important indicator of apoptosis, and cell membrane damage induced by viral infection (Figure 1D and E). HEV RNA was also detected in the infected cells by RT-PCR method (Figure 2A). Dot-blot results indicated the reactivity of anti-HEV specific antibody with the capsid proteins in the A549 cell (Figure 2B). Figure 2C indicated the unlabeled immune electron micrographs of HEV particles in the infected cells with a diameter about 30 nm. Electron micrograph revealed two morphology forms, one was the empty particle with no significant RNA-like density inside and another was the complete form, which consisted with previous report [22].

### 2-D Gel electrophoresis and mass spectrometry analysis

**Figure 3:**
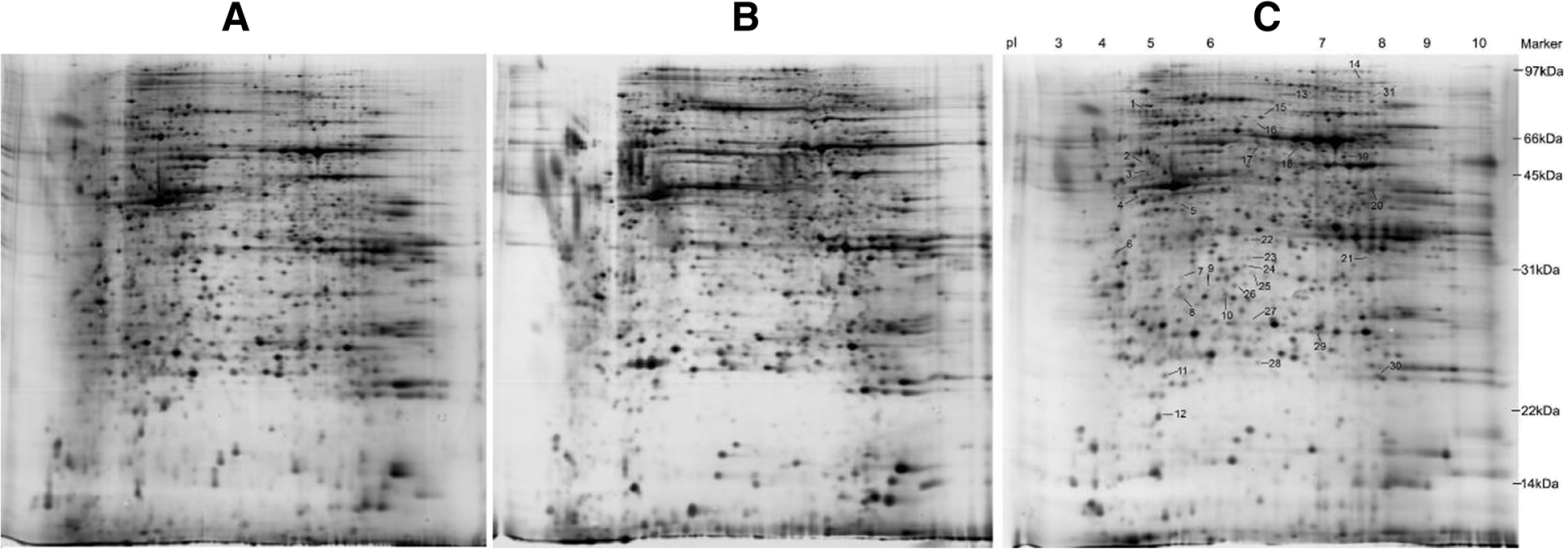
**2-DE analysis of the total proteins of control (A) and HEV infected A549 cell (B), and the protein dots with difference between the control and infected A549 cells (C).** Arrows indicated the isolated and identified protein spots.

To analyze the influence and changes in the cellular proteins of host cell with HEV infection, the cellular proteins in HEV-infected A549 and mock-infected cells were extracted for 2-DE analysis, respectively. To compensate the variability of gel electrophoresis, three independent 2-DE gels of cellular extracts from mock-infected or HEV-infected A549 cells were selected for statistical analysis (Figure 3).

**Figure 4:**
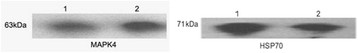
**Confirmation of two differentially expressed proteins, MAPK4 (left) and HSP70 (right) in control and HEV infected A549 cells by Western Blot assay.** 1: control A549 cells; 2: HEV infected A549 cells.

Of 2 585-3 152 protein spots visualized on each gel using silver staining, a total of 31 protein spots were found to be differentially expressed in HEV-infected A549 cells compared with mock-infected A549, including 10 significantly up-regulated protein spots and 21 significantly down-regulated protein spots (Figure 4). Six master gels representing post-infection (pi) and mock-infected cells were illustrated in Figure 4, and the location of the increased or decreased protein spots were marked by numbers.

A complete list of all these identified proteins during HEV infection with their protein score and sequence coverage was shown in Table 1.

**Table 1:** Identification of differentially expressed cellular proteins in HEV-infected A549 cells

**Spot no. (no. in 2-DE)**	**Protein name**	**Sequence coverage (%)**	**Accession no.**
2038 (22)	L-lactate dehydrogenase A-like	93	NP_001266265
2509 (27)	Triosephosphate isomerase	91	AEE79384
2352 (10)	Cathepsin D precursor	46	NP_599161
2755 (30)	manganese superoxide dismutase isoform B▲	90	NP_001019637
2556 (29)	Hypoxanthine-guanine phosphoribosyltransferase	83	NP_000185
1624 (20)	2-phosphopyruvate-hydratase alpha-enolase	94	CAA59331
2130 (21)	Carboxylesterase▲	79	AAB03611
2351 (8)	Chain B, Cathepsin D	84	P14091
1268 (18)	Chain A, Pyruvate Kinase M2 ▲	78	3H6O_B
714 (13)	Isoform 1 of Filamin-B	69	NP_001157789
891 (1)	FLNA filamin A,	60	BAJ83965
1770 (5)	Calponin-3 ▲	83	NP_001830
2245 (24)	Actin, cytoplasmic 1	71	NP_001092
2885 (12)	ACTA2 Actin, aortic smooth muscle	79	NP_001604
2754 (11)	Keratin, type I cytoskeletal 9	68	NP_000217
1400 (2)	26S protease regulatory subunit 6B▲	42	NP_006494
989 (15)	T-complex protein 1 subunit alpha	58	P17987
519 (14)	ATP-dependent RNA helicase	83	AAP97252
1267 (17)	Heterogeneous nuclear ribonucleoprotein H▲	69	NP_005511
1693 (4)	Heat shock-related 70 kDa protein 2	60	NP_068814
2328 (9)	PHB Prohibitin	49	P35232
1326 (19)	mitogen-activated protein kinase 9 ▲	68	AAH32539
1448 (3)	Mitogen-activated protein kinase 4▲	92	NP_002738
2302 (26)	NXA4 annexin IV	50	P09525
2702 (28)	Vacuolar ATP synthase catalytic subunit A	90	ADM12616
2193 (23)	annexin A1	88	NP_000691
2291 (25)	Proliferation-associated protein 2G4	90	NP_006182

Note: Black triangles indicated up-regulated proteins.

### Identification and functional classification of the differentially expressed proteins

To better understand the influence of cellular responses to HEV infection, these proteins were categorized with biological processes according to Uniprot knowledgebase.

#### (Swiss-Prot/TrEMBL) and gene ontology database

The identified cellular proteins were mainly involved in six groups including metabolism, cytoskeleton-associated protein, signal transduction, vesicular transporter, cell proliferation and lager molecular precessing. Interestingly, it was noted that four proteins (heat shock 27 kDa protein 1, 70 kDa heat shock cognate protein and proteasome beta 2, proteasome subunit alpha type 1) involved in stress response and ubiquitin-proteasome pathway, respectively, were both increased during PRRSV infection. Interestingly, it was noted that Heat shock-related 70 kDa protein 2 (HSPA2), which was associated with stress response, was increased during HEV infection, whereas, generally the expression of the member of HSP 70 were increased when cells are exposed to elevated temperatures or other stress [20],[23].

### Confirmation of proteomic data by western blot

Two identified proteins, MAPK4 for up-regulated expression and HSPA2 for down-regulated expression were selected to confirm by Western blot analysis, in which β-actin was used as an internal control. The results were consistent with the proteomic analysis of 2-DE gels as shown in Figure 1.

## Discussion

Our understanding of HEV has changed enormously over the past 30 years, from a waterborne infection causing outbreaks of acute hepatitis in developing countries to an infection of global distribution causing a range of hepatic and extra-hepatic illness [24]. To date, many unanswered questions including the zoonotic transmission, anti-virus treatment and pathogenetic mechanisms regarding HEV still remain. There is gathering evidence that HEV is enzootic and animals are considered of the reservoirs of human infection with this virus. However, the key proteins play important parts in the virus infection were still unknown. Understanding the changes of cellular proteins in these cells after exposed to HEV is helpful for elucidating molecular mechanism associated with functional alterations of HEV-infected susceptible cells. In the present study, we for the first time applied proteomic method to identify the differentially expressed cellular proteins of A549 cells during infection in vitro. HEV was divided into at least four genotypes, among which genotype 3 and 4 belong to zoonotic pathogens. Hence, a genotype 4 stain isolated from China was used to infect A549 cell. The changes in the cellular proteins of A549 infection or not were identified by combination of 2-DE and MS method. From our present data, 31 cellular proteins expressed differentially were identified uniquely and the functional roles of the proteins of interest exposed to HEV infection were discussed as follows.

HSPA2 is a member of heat shock protein 70 (HSP70) family, which has important intracellular functions including folding of nascent polypeptide chains, translocation of proteins across membranes, intracellular vesicle trafficking and sorting, uncoating of clathrin-coated vesicles, signal transduction, and nuclear transport [25],[26]. Previous studies indicated that HSP70 accelerate and facilitate the virus propagation. Our results indicated that HSPA2 were decreased when cells exposed to HEV. We speculate that cells decrease the expression of HSPA2 by themselves to protect them from infection and damage from HEV.

One up-regulated protein of interest in the present study was Heterogeneous nuclear ribonucleoprotein H (HNRPH1), a member of Heterogeneous nuclear ribonucleoproteins (hnRNPs) which are complexes of RNA and protein present in the cell nucleus during gene transcription and subsequent post-transcriptional modification of the newly synthesized RNA (pre-mRNA). The presence of the proteins bound to a pre-mRNA molecule serves as a signal that the pre-mRNA is not yet fully processed and ready for export to the cytoplasm. Since most mature RNA is exported from the nucleus relatively quickly, most RNA-binding proteins in the nucleus exist as heterogeneous ribonucleoprotein particles. After splicing has occurred, the proteins remain bound to spliced introns and target them for degradation. Hahm et al found that heterogeneous nuclear ribonucleoprotein L (hnRNP L) interacts with the 3′ border of the internal ribosomal entry site of hepatitis C virus, and the binding of hnRNP L to the HCV IRES correlates with the translational efficiencies of corresponding mRNAs [27]. In addition, another hnRNPs protein, hnRNP A1, was identified to facilitate hepatitis C virus replication [28]. In the current study, hnRPH1 was up-regulated during infection with HEV, which suggested that play a part in the HEV mRNA translation, processing or degradation. The exact role that hnRPH1 paly in the HEV infection will be identified in the futher research.

Taken together, our work is the first time regarding the proteomic analysis on the cellular responses to HEV infection. Thirty-one cellular proteins expressed differentially were identified unambiguously. These data are helpful for investigating the molecular basis associated with the interaction between HEV and the host cells although more efforts should be required to discover the mechanisms.

## Conclusions

Taken together, a total of 31 protein spots were found to be differentially expressed in HEV-infected A549 cells compared with mock-infected A549, including 10 significantly up-regulated protein spots and 21 significantly down-regulated protein spots. Although, the proteins that play important part in the infection of HEV were not identified, the present study will lay a foundation for further research of HEV infection.

## Competing interests

The authors declare that they have no competing interests.

## Authors’ contributions

QS and WZ conceived the study and designed the experiments. YYP, XLF, YX, XBB conducted the experiments. QS, SXY drafted the final manuscript with the help of LC. YY, XCW and HW helped in designing the experiments and provided samples and reagents for the study. All authors read and approved the final manuscript.
